# Letter to the editor regarding: ‘increased purchases of prescription medicines in offspring of women with type 1 diabetes: a Finnish register-based cohort study between 1995 and 2018’

**DOI:** 10.1080/07853890.2024.2447401

**Published:** 2024-12-31

**Authors:** Yao Yin, Qing Lan, Si Jin

**Affiliations:** a Department of Endocrinology, Institute of Geriatric Medicine, Liyuan Hospital, Tongji Medical College, Huazhong University of Science and Technology, Wuhan, China; b Department of Gastroenterology, Liyuan Hospital, Tongji Medical College of Huazhong University of Science and Technology, Wuhan, China

We read with great interest the article by Korpijaakko et al. [1], titled ‘Increased purchases of prescription medicines in offspring of women with type 1 diabetes: a Finnish register-based cohort study between 1995 and 2018’’, published in your journal *Annals of Medicine*. This study is commendable for its substantial sample size, diverse ethnic representation, and extensive follow-up period, providing valuable insights into the long-term effects of maternal type 1 diabetes on offspring health. The longitudinal follow-up of up to 23 years significantly enhances the robustness of these findings. However, several limitations warrant further discussion to strengthen the conclusions drawn.

Firstly, the study’s focus on specific regions in Finland, characterized by well-developed medical resources and social welfare systems, raises questions about the generalizability of the results to other countries or regions. Future research should incorporate multicentre data from various geographical locations to validate the applicability of these findings across different healthcare contexts.

Secondly, while the investigation centers on the health impacts of type 1 diabetes, the absence of strict control for other health conditions among participants is a notable limitation. Individuals with type 1 diabetes often experience comorbidities, such as asthma and thyroid disorders [2]. Consequently, the offspring in the exposed group may inherit multiple health issues from their mothers, potentially leading to an inflated number of prescription drug purchases, which could confound the results. Furthermore, although the study matched mothers by age and region, it did not account for socioeconomic factors (e.g. income, education, occupation), which are closely related to healthcare utilization and may influence prescription drug purchasing behaviours [3].

Additionally, while the study estimates prescription drug use *via* Defined Daily Doses (DDD) per person-year, this method is based on adult standards. Given that many participants were minors during the follow-up period, this could lead to inaccuracies in estimating medication use. Adjustments based on paediatric dosing guidelines would provide a more accurate representation of actual drug utilization.

The authors acknowledge the potential clustering of observational data and the higher frequency of second-degree relatives in the exposed group. It is advisable to employ appropriate statistical methods, such as mixed effects models or hierarchical regression analysis, to account for this clustering effect and enhance the accuracy of the findings. Moreover, several potential confounders [4]—including maternal mental health, weight changes, medication use, lifestyle factors, dietary habits, and parenting styles—were not considered. These factors could significantly influence the results, particularly given the established association between maternal depression and type 1 diabetes [5].

In conclusion, while Korpijaakko et al.’s study lays an important foundation for understanding the implications of maternal health during pregnancy on offspring outcomes, addressing the aforementioned limitations could further strengthen the evidence base and improve the clinical applicability of the findings. We commend the authors for their significant contribution to this vital area of research and look forward to future studies that build upon this work.

## Data Availability

Data sharing is not applicable to this article as no new data were created or analyzed in this study.
